# Quantitative Estimation of the Climatic Effects of Carbon Transferred by International Trade

**DOI:** 10.1038/srep28046

**Published:** 2016-06-22

**Authors:** Ting Wei, Wenjie Dong, John Moore, Qing Yan, Yi Song, Zhiyong Yang, Wenping Yuan, Jieming Chou, Xuefeng Cui, Xiaodong Yan, Zhigang Wei, Yan Guo, Shili Yang, Di Tian, Pengfei Lin, Song Yang, Zhiping Wen, Hui Lin, Min Chen, Guolin Feng, Yundi Jiang, Xian Zhu, Juan Chen, Xin Wei, Wen Shi, Zhiguo Zhang, Juan Dong, Yexin Li, Deliang Chen

**Affiliations:** 1 State Key Laboratory of Severe Weather, Chinese Academy of Meteorological Sciences, Beijing 100081, China; 2 School of Atmospheric Sciences, Sun Yat-Sen University, Guangzhou 510275, China; 3 Zhuhai Joint Innovative Center for Climate-Environment-Ecosystem, Future Earth Research Institute, Beijing Normal University, Zhuhai 519087, China; 4 CAS Center for Excellence in Tibetan Plateau Earth Sciences, Beijing 100101, China; 5 State Key Laboratory of Earth Surface Processes and Resource Ecology, Beijing Normal University, Beijing 100875, China; 6 College of Global Change and Earth System Science, Beijing Normal University, Beijing 100875, China; 7 Arctic Centre, University of Lapland, PL122, 96100 Rovaniemi, Finland; 8 Nansen-Zhu International Research Centre, Institute of Atmospheric Physics, Chinese Academy of Sciences, Beijing 100029, China; 9 Marine Weather Forecast Division, National Marine Environmental Forecasting Center, Beijing 100081, China; 10 The State Key Laboratory of Numerical Modelling for Atmospheric Sciences and Geophysical Fluid Dynamics, Institute of Atmospheric Physics, Chinese Academy of Sciences, Beijing, 100029, China; 11 Institute of Space and Earth Information Science, Chinese University of Hong Kong, HKSAR, China; 12 School of Geographic Science, Nanjing Normal University, Nanjing 210023, China; 13 National Climate Center, China Meteorological Administration, Beijing 100086, China; 14 Zhuhai Meteorological Bureau, Zhuhai 519000, China; 15 Department of Earth Sciences, University of Gothenburg, Box 460, S-405 30 Gothenburg, Sweden

## Abstract

Carbon transfer via international trade affects the spatial pattern of global carbon emissions by redistributing emissions related to production of goods and services. It has potential impacts on attribution of the responsibility of various countries for climate change and formulation of carbon-reduction policies. However, the effect of carbon transfer on climate change has not been quantified. Here, we present a quantitative estimate of climatic impacts of carbon transfer based on a simple CO_2_ Impulse Response Function and three Earth System Models. The results suggest that carbon transfer leads to a migration of CO_2_ by 0.1–3.9 ppm or 3–9% of the rise in the global atmospheric concentrations from developed countries to developing countries during 1990–2005 and potentially reduces the effectiveness of the Kyoto Protocol by up to 5.3%. However, the induced atmospheric CO_2_ concentration and climate changes (e.g., in temperature, ocean heat content, and sea-ice) are very small and lie within observed interannual variability. Given continuous growth of transferred carbon emissions and their proportion in global total carbon emissions, the climatic effect of traded carbon is likely to become more significant in the future, highlighting the need to consider carbon transfer in future climate negotiations.

Humans have for centuries been changing the composition of the Earth’s atmosphere, leading to significant climate change and air pollution, but the process has been particularly rapid since the 1950s^1^. To avoid the serious threat to the environment posed by exponential growth of greenhouse gas emissions, the international community has tried for 20 years to reduce global carbon emissions through sovereign state-level negotiations. One critical issue in these negotiations is to differentiate the historical responsibility for climate change and make a fair emission reduction program for different countries. Previous attribution studies of responsibility for climate change^2^,^3^,^4^,^5^,^6^ have been based on production-based emissions, i.e., accounted for using territorial boundaries^7^. These production-based emissions allow for convenient monitoring and regulation. However, international trade creates a geographic separation between the product’s final consumers and the carbon emitted in the production process, effectively shifting the CO_2_ associated with their consumption to distant lands^8^,^9^,^10^,^11^. This challenges the traditional principle of “the polluter pays”. One way of rectifying this problem is that responsibilities for climate change should be attributed in accordance with consumption-based accounting of carbon emissions that is defined as adding the emissions associated with imports and subtracting the emissions associated with exports, from production-based emissions^12^,^13^,^14^. Therefore, it has been argued that the current production-based carbon emission inventories should be replaced by consumption-based system in formulating emission reduction policies in post-Kyoto frameworks^15^,^16^,^17^.

Carbon emissions embodied in international trade rose by ~38% from 1990 to 2008^18^, and the trend has continued in recent years^11^,^19^,^20^,^21^, motivating our quantification of its impact on the climate change attribution and responsibilities for mitigation. We present estimates of the role of carbon emissions in international trade using both a simple model (allowing calculations over a longer time interval) and three state-of-the-art Earth System Models (limited to a shorter study period by data availability), and explore the potential impact of transferred carbon emissions on the Kyoto Protocol (KP)^22^. We believe that the results will be useful for international negotiations in the future.

## Results and Discussions

**Figure 1:**
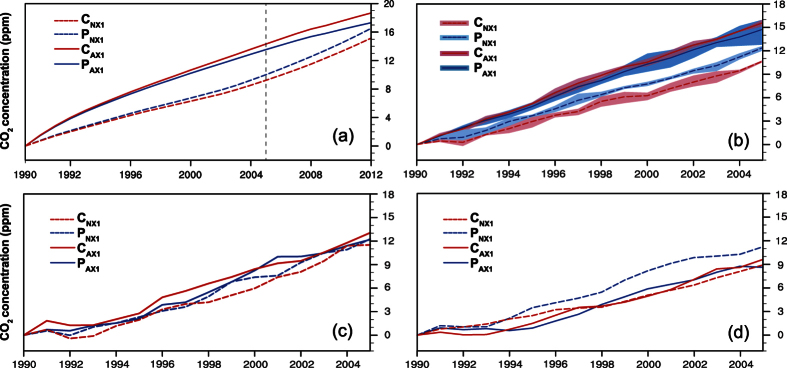
Temporal evolution of the simulated atmospheric CO_2_ concentration changes relative to 1990 using (**a**) IRF, (**b**) CESM, (**c**) BNU-ESM and (**d**) FGOALS-s2 under the P_AX1_, P_NX1_, C_AX1_ and C_NX1_ scenarios. (**b**) Shading shows the range of CO_2_ changes due to different initial conditions and lines are the ensemble mean.

Carbon emissions via international trade potentially reduce the gap in historical responsibilities for CO_2_ loading between developed and developing countries. To investigate the influence of transferred carbon on historical responsibility for climate change, four experiments were designed and executed with a simple CO_2_ Impulse Response Function (IRF) model and three Earth System Models (Methods). The experiments are (i) P_AX1_: production-based CO_2_ emissions only allowed from developed countries (i.e., Annex I countries); (ii) P_NX1_: production-based CO_2_ emissions only allowed from developing countries (i.e., Non-annex I countries); (iii) C_AX1_: consumption-based CO_2_ emissions only allowed from the developed countries; and (iv) C_NX1_: consumption-based CO_2_ emissions only allowed from the developing countries. The simulations show that atmospheric CO_2_ concentrations would increase by 8.6–14.8 ppm (11.2–12.5 ppm) from 1990 to 2005 on conditions that production-based CO_2_ emissions are only allowed from the developed (developing) countries (Fig. 1). If consumption-based CO_2_ emissions are only allowed from the developed countries or the developing countries, atmospheric CO_2_ concentrations show an increase of 9.6–15.6 ppm and 8.9–11.5 ppm, respectively. Therefore, over the period 1990–2005, 0.8–2.3 ppm CO_2_ was transferred from the developed world to the developing world via international trade. This indicates that 3–9% of responsibility for the increased atmospheric CO_2_ concentration was shifted from the developed countries to the developing countries between 1990 and 2005 based on the normalized proportional method^5^(Table 1). These results suggest that transferred carbon emissions reduce the difference in historical responsibilities for CO_2_ loading between the developed and the developing countries, though these amounts are small.

Over the longer period (1990–2012), carbon emissions via international trade resulted in an increase of CO_2_ by ~1.4 ppm and hence a shift of historical responsibility by ~4% based on the IRF model. These numbers are quite similar to results for the 1990–2005 period from the Earth System Models. It should be noted that transferred carbon emissions account for a considerable proportion in production-based emissions for some regions and countries (e.g., China, USA and EU28). Based on the IRF model, carbon transfer (1990−2012) leads to a migration of CO_2_ by ~1.08 ppm (accounting for ~17.2% of CO_2_ rise that results from consumption-based emissions) from other countries to China, whereas a transfer of CO_2_ by ~0.33 and 1.17 ppm (4.6% and 19.3%) from USA and EU28 to other countries, respectively (Supplementary Fig. S1).

**Figure 2:**
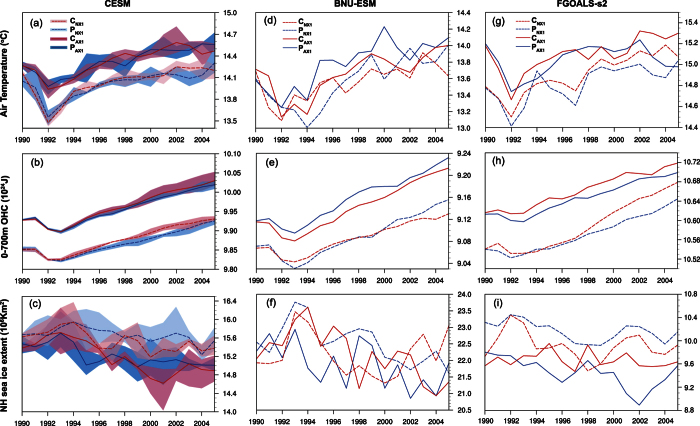
Temporal evolution of annual mean surface air temperature, upper ocean heat content (0–700 m) and Northern Hemisphere sea ice fraction simulated by CESM (left panel), BNU-ESM (middle panel), and FGOALS-s2 (right panel) under the P_AX1_, P_NX1_, C_AX1_ and C_NX1_ scenarios. Left panel: shading shows the range of values due to different initial conditions and lines are the ensemble mean.

As may be expected given the relatively small levels of CO_2_ involved, the climate system shows little response to the carbon transferred via international trade. The modeled warming of global atmosphere and oceans, and the melting of sea-ice in Northern Hemisphere are similar under all scenarios between 1990–2005 (Fig. 2); also borne out by differences in initial conditions being comparable with differences between the experiments (Figs 1 and 2). If a longer history of trade was available then climate effects due to trade may be more discernable, although trade has only grown rapidly in recent decades. The amount of transferred carbon emissions and their proportion in global total carbon emissions are gradually increasing^18^,^19^,^21^, and so traded carbon is likely to become more significant in future.

**Figure 3:**
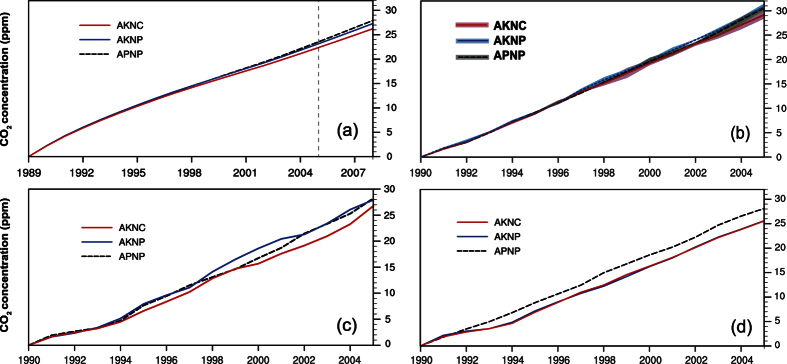
Same as in Fig. 1 but under the APNP, AKNP, and AKNC scenarios.

**Figure 4:**
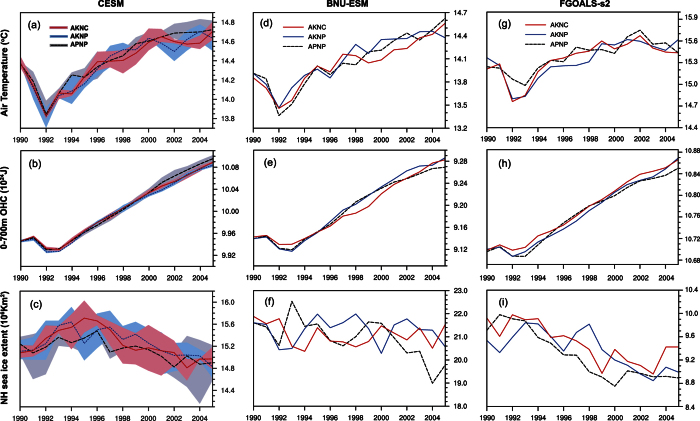
Same as in Fig. 2 but under the APNP, AKNP, and AKNC scenarios.

Transferred carbon emissions will, to an extent, affect the effectiveness of the Kyoto Protocol. To investigate the impact of transferred carbon on the KP, we construct three CO_2_ emission pathways for 1990–2005 depending on whether carbon transfers are allowed between the developed and the developing countries while following CO_2_ mitigation protocols in KP (Methods). Under the scenario that the developed and the developing countries ignore their pledges and follow their production-based emissions (APNP; equivalent to the CMIP5 *historical* experiment), the simulated CO_2_ concentration in 2005 is 23.5–30.6 ppm higher than in 1990 (Fig. 3). If the developed countries follow the KP and the developing countries pursue their production-based emission (AKNP; as is specified by the KP), the increase of CO_2_ is simulated as 23.2–30.8 ppm. Therefore, actual global carbon emissions are seemingly in keeping with the KP. When the developed countries follow the KP and the developing countries pursue their consumption-based emission (AKNC; equivalent to the KP without counting carbon transfers from the developed to the developing countries), simulated CO_2_ concentration increases by 22.4–29.2 ppm, 0–1.6 ppm less than that simulated by AKNP. We now define the relative change of CO_2_ concentration under a mitigation scenario (i.e. AKNP or AKNC) to that under the observed emission scenario (i.e. APNP) as the effectiveness of the mitigation scenario. These 1990–2005 simulations indicate that the effectiveness of AKNP and AKNC is −0.7–8.9% and 4.6–8.9%, respectively (Table 1). This result indicates that the trade between the developed countries and the developing world contributed up to 5.3% of CO_2_ concentration increases from 1990–2005 (Table 1). This is the contribution to CO_2_ rise from items actually used in the developed world but which were produced in the developing world, and hence escaped the limitations of the KP. Over the whole first commitment period of KP (1990–2008) simulated by the IRF model, 3.7% of CO_2_ increase can be similarly attributed. The accumulated sum of transferred emissions (0–1.6 ppm) from 1990 to 2005 is, however, small: less than the annual increase of CO_2_ (~1.7 ppm/yr) over the same period. The climate system hence shows little response to the transferred emissions (Fig. 4). Overall, the effectiveness of the Kyoto Protocol may have been potentially increased during 1990–2005 if the transferred carbon emissions are taken into account, though the resulting CO_2_ concentrations reduction and climate responses are tiny.

Numerical simulations with the IRF and three Earth System Models reveal that including carbon in international trade reduces the gap of historical responsibilities between the developed and the developing countries and the effectiveness of the KP. Although the climate change caused by the transferred carbon emissions (1990−2005) is almost negligible, the climatic effects of embodied emissions is expected to be more profound in future as global trade appears set to continue to grow. International trade also results in transfer of polluting gases which has additional environment and health hazards to the regions where goods are produced. For example, we estimate that the developed countries transferred 2.26 teragrams of SO_2_ to the developing world in 1990, which grew to 3.28 teragrams by 2005 (Supplementary Fig. S2). In addition, international trade potentially increases global carbon emissions as carbon-intensive manufacturing in emerging countries (e.g., China) entails more carbon emissions than would making the same product in the developed (importing) countries^23^. Given continuous growth of transferred carbon emissions and likely more significant impact on climate change, future climate negotiations should take into consideration embodied emissions in international trade. This entails accurate national carbon emissions accounting^24^ and implementation of incentives to make a feasible, fair emissions reduction policy.

It is undeniable that international trade affects global carbon emissions, air pollution and countries historical responsibility by redistributing emissions related to production of goods and services. But countries with net exports profit while bearing the extra climatic and environmental burden. Whether the profits compensate for the damage, especially over the long run, is still an open question which has many other dimensions and cannot be properly addressed by simple measurement or models.

## Methods

### Model Description

We use a CO_2_ Impulse Response Function (IRF) and three Earth System Models that have participated in the Coupled Model Intercomparison Project Phase 5 (CMIP5). The IRF is used to calculate CO_2_ concentration by a sum of exponentially decaying functions, one for each fraction of the additional concentrations, which should reflect the time scales of different sinks^25^.


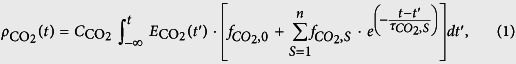


where 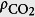 is CO_2_ concentrations, 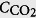 is a constant and set to approximately 0.47 ppmv/GtC, 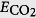 is the emission of CO_2_, 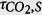 is the atmospheric exponential decay time of the s^th^ fraction of the additional concentration CO_2_ (171.0, 18.0 and 2.57 years), 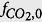 is the first fraction (0.152), and 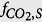 is the respective fraction (0.253, 0.279 and 0.316). The coefficients are based on the impulse response of the Bern model^26^ as used in the IPCC–SAR and IPCC–TAR.

The three Earth System Models are the Community Earth System Model (CESM)^27^, the Beijing Normal University-Earth System Model (BNU-ESM)^28^ and the Flexible Global Ocean-Atmosphere-Land System model (FGOALS-s2)^29^. Each of the three Earth System Models contains an interactive carbon cycle module in the land component and an ecosystem-biogeochemical module in the ocean component. The simulated atmospheric CO_2_ concentrations are fully coupled to the land and ocean surface CO_2_ fluxes and are used directly to compute the radiative forcing, hence forming a complete carbon cycle process. In this study, the atmospheric horizontal resolution of the CESM, BNU-ESM and FGOALS-s2 is ~0.9° × 1.25°, ~2.8° × 2.8°, and ~2.81° × 1.66°, respectively. The ocean component has a nominal 1° resolution for the CESM and 1° × 1° for the BNU-ESM and FGOALS-s2.

### Experimental design

Two groups of numerical experiments were designed to investigate the influence of transferred carbon emissions. In group I, the influence of transferred carbon emissions on historical climate change is examined. We design four scenarios in which production-based/consumption-based emissions are allowed only from either the developed countries or the developing countries (Supplementary Table S1). The production-based carbon emissions fluxes are available at 1° × 1° spatial resolution from 1751 to 1949 at annual resolution and from 1950 to 2007 at monthly resolution^30^. The national inventories of consumption-based carbon emissions^18^ cover 113 regions and extend from 1990 to 2008. We use the regional distribution of production-based carbon fluxes to construct gridded consumption-based carbon fluxes at monthly and 1° × 1° spatial resolution. The cumulative transferred carbon emissions in the developed and the developing countries are shown in Supplementary Fig. S3. The CESM was first integrated over the period of 1850–1990 under the P_AX1_ and P_NX1_ scenarios (Supplementary Table S1), respectively. Initialized from the year at 1990 in the P_AX1_ (P_NX1_) experiment, CESM was further run from 1990 to 2005 under the P_AX1_ and C_AX1_ (P_NX1_ and C_NX1_) scenarios. Other forcings varying over the historical period (1850–2005) include CH_4_, N_2_O, halocarbons, aerosols, solar irradiance, and volcanoes. The same method is used for the BNU-ESM and FGOALS-s2. Note that we run all the sensitivity experiments (groups I and II) with three different initial conditions using CESM. For the IRF, we construct four time series of carbon emissions (1850–2012) based on the designed emissions scenarios (Supplementary Table S1).

In group II, the effect of mitigation of production-based and consumption-based counting on the KP is investigated. We assume that each developed country decreases (or increases) its annual carbon emissions linearly and achieves its reduction commitment in 2008 according to the KP—whose purpose is reducing the overall emissions of anthropogenic greenhouse gases of the developed world by at least 5% below the 1990 levels in the commitment period from 2008 to 2012. We construct three emission inventories (Supplementary Table S1) for each developed country from 1990 to 2005 at monthly and 1° × 1° spatial resolution (Supplementary Fig. S4). For the CESM, BNU-ESM, and FGOALS-s2, each model is first integrated over the period of 1850–1990 under the scenario that all countries follow their production-based carbon emissions (equivalent to the CMIP5 *historical* experiment). Starting from the end of this experiment, each model was then run from 1990 to 2005 under the APNP, AKNP and AKNC scenarios (Supplementary Table S1). For the IRF, we create three time series of carbon emissions (1850–2008) based on the designed emissions pathways (Supplementary Table S1).

## Additional Information

**How to cite this article**: Wei, T. *et al*. Quantitative Estimation of the Climatic Effects of Carbon Transferred by International Trade. *Sci. Rep.* **6**, 28046; doi: 10.1038/srep28046 (2016).

## Supplementary Material

Supplementary Information

## Figures and Tables

**Table 1:** Contributions of the developed (AX1) and developing (NX1) countries to the rise in atmospheric CO_2_ concentration and the effectiveness of AKNP and AKNC scenarios (see text for definition).

	IRF^a^	CESM^a^	BNU-ESM^a^	FGOALS^a^	IRF^b^
AX1 production-based contribution	57%	53%	50%	43%	55%
NX1 production-based contribution	43%	47%	50%	57%	45%
AX1 consumption-based contribution	60%	59%	53%	52%	51%
NX1 consumption-based contribution	40%	41%	47%	48%	49%
Transferred contribution	3%	6%	3%	9%	4%
Effectiveness of AKNP	1.7%	−0.7%	1.1%	8.9%	2.3%^c^
Effectiveness of AKNC	5.0%	4.6%	5.2%	8.9%	6.0%^c^
Transferred effectiveness	4.3%	5.3%	4.1%	0%	3.7%^c^

^a^From 1990 to 2005.

^b^From 1990 to 2012.

^c^From 1990 to 2008.
