# Supplementary material for: Quantitative Estimation of the Climatic Effects of Carbon Transferred by International Trade
Source: Sci Rep. 2016 Jun 22;6:28046. doi: 10.1038/srep28046 (PMC4916407; doi:10.1038/srep28046)
Supplement: Supplementary Information [file srep28046-s1.pdf]

Supplementary Information for

Quantitative Estimation of the Climatic Effects of Carbon Transferred by International Trade

Ting Wei, Wenjie Dong, John Moore, Qing Yan, Yi Song, Zhiyong Yang, Wenping Yuan, Jieming Chou, Xuefeng Cui, Xiaodong Yan, Zhigang Wei, Yan Guo, Shili Yang, Di Tian, Pengfei Lin, Song Yang, Zhiping Wen, Hui Lin, Min Chen, Guolin Feng, Yundi Jiang, Xian Zhu, Juan Chen, Xin Wei, Wen Shi, Zhiguo Zhang, Juan Dong, Yexin Li, and Deliang Chen

Contents of this file

- Estimation of SO_2 emissions via international trade
- Table S1. Summary of emissions scenarios in this study.
- Figure S1. (a) Transferred carbon emissions (TCE; solid lines; left axis) and their proportion in production-based emissions (dash lines; right axis) for China, USA and EU28. Positive (negative) value represent a net exporter (importer) of embodied emissions. (b) Simulated atmospheric CO_2 concentration changes relative to 1990 due to production/consumption-based emissions for China, USA and EU28 using a CO_2 Impulse Response Function. Data source: global carbon budget (http://www.globalcarbonproject.org/carbonbudget/).
- Figure S2. Time series of production-based SO_2 emissions (thin solid lines), consumption-based SO_2 emissions (thick solid lines), column integrated optical depth due to sulfate at 550nm (dots), and content of PM10 (dash lines) in the developed (red) and the developing (blue) countries.
- Figure S3. Cumulative CO_2 emission flux (Kg/m^2/year) from 1990 to 2005 for (a) developed world consumption-based emission and (c) its anomalies relative to the production-based emission, and (b) developing world consumption-based emission and (d) its anomalies relative to the production-based emission. The maps were created using NCAR Command Language Version 6.3.0 (http://dx.doi.org/10.5065/D6WD3XH5).
- Figure S4. (a) Time series of CO_2 emissions from the developed countries based on production, consumption and KP. (b) Time series of CO_2 emissions under the APNP, AKNP, and AKNC scenarios. (c) Cumulative CO_2 emission flux (Kg/m^2/year) difference between the AKNP and APNP scenarios from 1990 to 2005. (d) Cumulative CO_2 emission flux (Kg/m^2/year) difference between the AKNC and APNP scenarios from 1990 to 2005. The maps were created using NCAR Command Language Version 6.3.0 (http://dx.doi.org/10.5065/D6WD3XH5).

Estimation of SO_2 emissions via international trade

CO_2 is just one of many greenhouse gases emitted in the production of goods and services. Other greenhouse gases, and pollutants are also transferred along with CO_2 via international trade[1,2]. We use the Long-Term Consumption Measurement model[3] to calculate the consumption-based and transferred SO_2 emissions of each country during 1990–2005 via international trade:

$$F_{\mathrm{Cr}}(r,i) = F_{\mathrm{Pr}}(r,i) + \mathrm{COEF}_{im}(i) * \mathrm{Imports}(r,i) - \mathrm{COEF}(r,i) * \mathrm{Exports}(r,i)$$
$$\sum_r F_{\mathrm{Cr}}(r,i) = \sum_r F_{\mathrm{Pr}}(r,i)$$

where $F_{\mathrm{Cr}}(r,i)$ and $F_{\mathrm{Pr}}(r,i)$ is the consumption-based and production-based SO_2 emissions ($PgSO_2$) for region *r* in year *i*, respectively. $\mathrm{Imports}(r,i)$ and $\mathrm{Exports}(r,i)$ are the annual trade of goods and services from each region *r*. $\mathrm{COEF}(r,i)$ is the production intensity of SO_2 (SO_2 emissions per unit of Gross Productive Output). $\mathrm{COEF}_{im}(i)$ is the importation intensity of SO_2 (SO_2 emissions per unit of importation) and a global averaged value is assigned. The constraints reveal that total production-based SO_2 emissions in a specific year must equal those of the consumption-based emissions in the same year over all countries. Input data is production-based SO_2 emission data[4], Gross Domestic Product and imports[5], trade volume of goods and service[6], and the balance of payments current account[7] for the period of 1990–2005.

We estimate that the developed countries transferred 2.26 teragrams of SO_2 (accounting for 1.9% of the global SO_2 emissions) to the developing world in 1990, which grew to 3.28 teragrams (3.1%) by 2005 (Fig. S2). Some anthropogenic SO_2 is oxidized to sulfuric acid and sulfate aerosol[8]; those with diameters of 10 μm or less (PM10), have unfavorable impacts on the local environment and human health. Trends in anthropogenic

SO_2 emissions, sulfate aerosol[9] and PM10[10] in the developed countries have decreased during 1990 to 2005, while there has been an increasing trend in the developing countries. PM10 and production-based SO_2 emissions in the developing countries are significantly positively correlated (detrended correlation coefficient = 0.53, $p<0.05$). Hence, the transfer of polluting gases via international trade has additional environment and health hazards to the regions where goods are produced that are difficult to cost, especially in the long term.

References

1. Kanemoto, K., Moran, D., Lenzen, M. & Geschke, A. International trade undermines national emission reduction targets: New evidence from air pollution. Glob. Environ. Change 24, 52–59 (2014).
2. Lin, J. et al. China's international trade and air pollution in the United States. Proc. Natl. Acad. Sci. USA 111, 1736–1741 (2014).
3. Yang, Z. et al. Constructing long-term (1948–2011) consumption-based emissions inventories. J. Clean Prod. 103, 793–800 (2014).
4. Smith, S. J. et al. Anthropogenic Sulfur Dioxide Emissions: 1850–2005. Atmos. Chem. Phys. 11, 1101–1116 (2011).
5. World Bank. *GDP and Imports of Goods and Services in Current U.S. Dollars (World Development Indicators).* (2014) Available at: http://data.worldbank.org/products/wdi. (Date of access: 1st January 2015).

6. World Trade Organization. *Time Series on International Trade.* (2014) Available at: http://stat.wto.org/StatisticalProgram/WSDBStatProgramHome.aspx?Language¼E. (Date of access: 1st January 2015).

7. International Monetary Fund. *Balance of Payments version 5 & 6*. (2014) Available at: http://elibrary-data.imf.org/DataExplorer.aspx. (Date of access: 1st January 2015).

8. Gray, B. A. et al. Sources, transport, and sinks of SO2 over the equatorial Pacific during the Pacific Atmospheric Sulfur Experiment. J. Atmos. Chem. 68, 27–53 (2011).

9. Chin, M., Savoie, D., Thornton, D., Bandy, A. & Huebert, B. Atmospheric sulfur cycle inthe global model GOCART: Comparison with observations. J. Geophys. Res. 105, 24,698–24,712 (2000).

10. Emission Database for Global Atmospheric Research (EDGAR). *Global Emissions EDGAR v4.2*. (2011) Available at: http://edgar.jrc.ec.europa.eu/overview.php?v=42. (Date of access: 1st January 2015).

11. The NCAR Command Language (Version 6.3.0) [Software]. Boulder, Colorado: UCAR/NCAR/CISL/TDD. http://dx.doi.org/10.5065/D6WD3XH5 (2016).

Table S1. Summary of emissions scenarios in this study.

Scenarios		Carbon emissions	
		developed countries	developing countries
Group I	P_{AX1}	production-based emissions	zero emissions
	C_{AX1}	consumption-based emissions	zero emissions
	P_{NX1}	zero emissions	production-based emissions
	C_{NX1}	zero emissions	consumption-based emissions
Group II	APNP	production-based emissions	production-based emissions
	AKNP	follow KP emissions	production-based emissions
	AKNC	follow KP emissions	consumption-based emissions

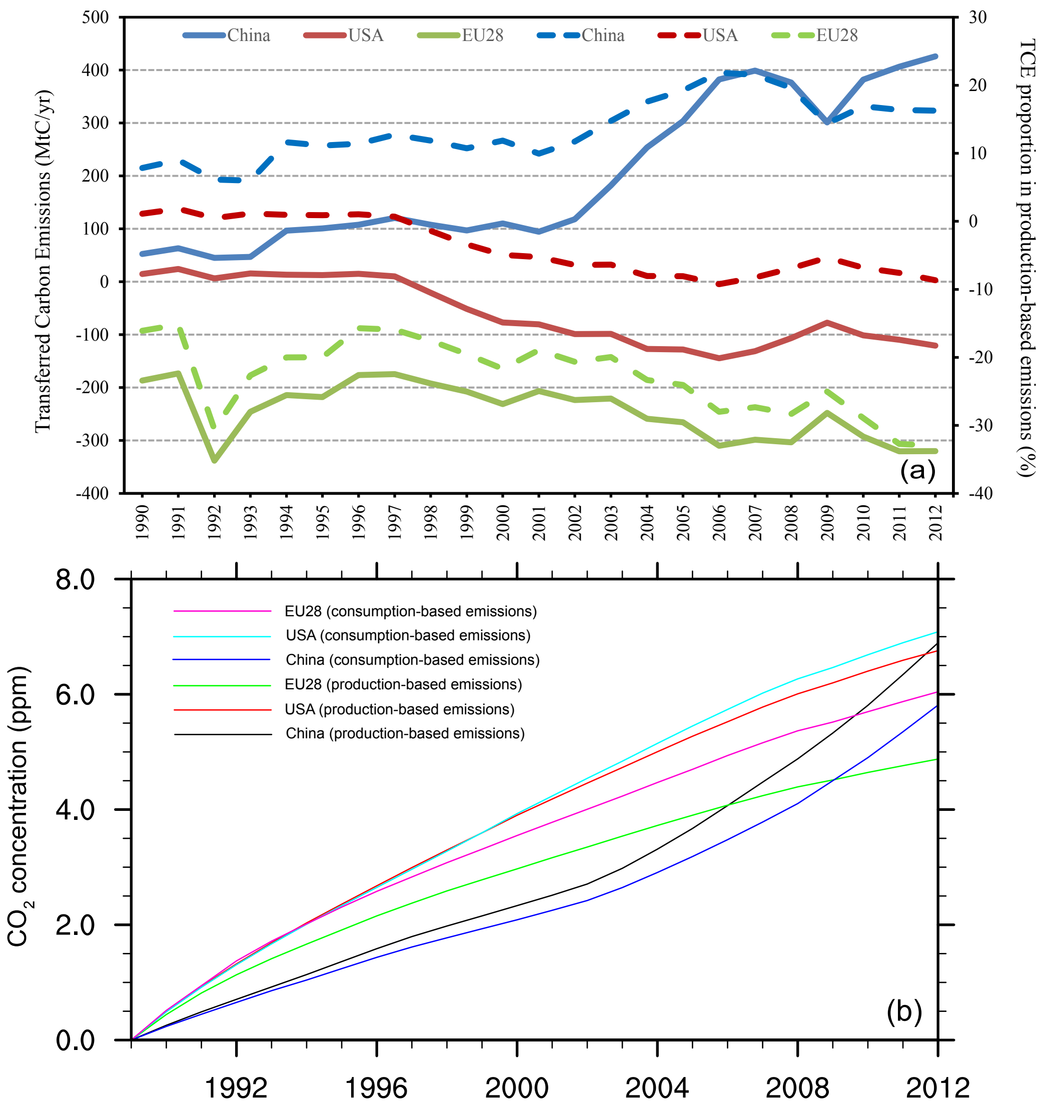

Figure S1. (a) Transferred carbon emissions (TCE; solid lines; left axis) and their proportion in production-based emissions (dash lines; right axis) for China, USA and EU28. Positive (negative) value represent a net exporter (importer) of embodied emissions. (b) Simulated atmospheric CO_2 concentration changes relative to 1990 due to production/consumption-based emissions for China, USA and EU28 using a CO_2 Impulse Response Function. Data source: global carbon budget (http://www.globalcarbonproject.org/carbonbudget/)

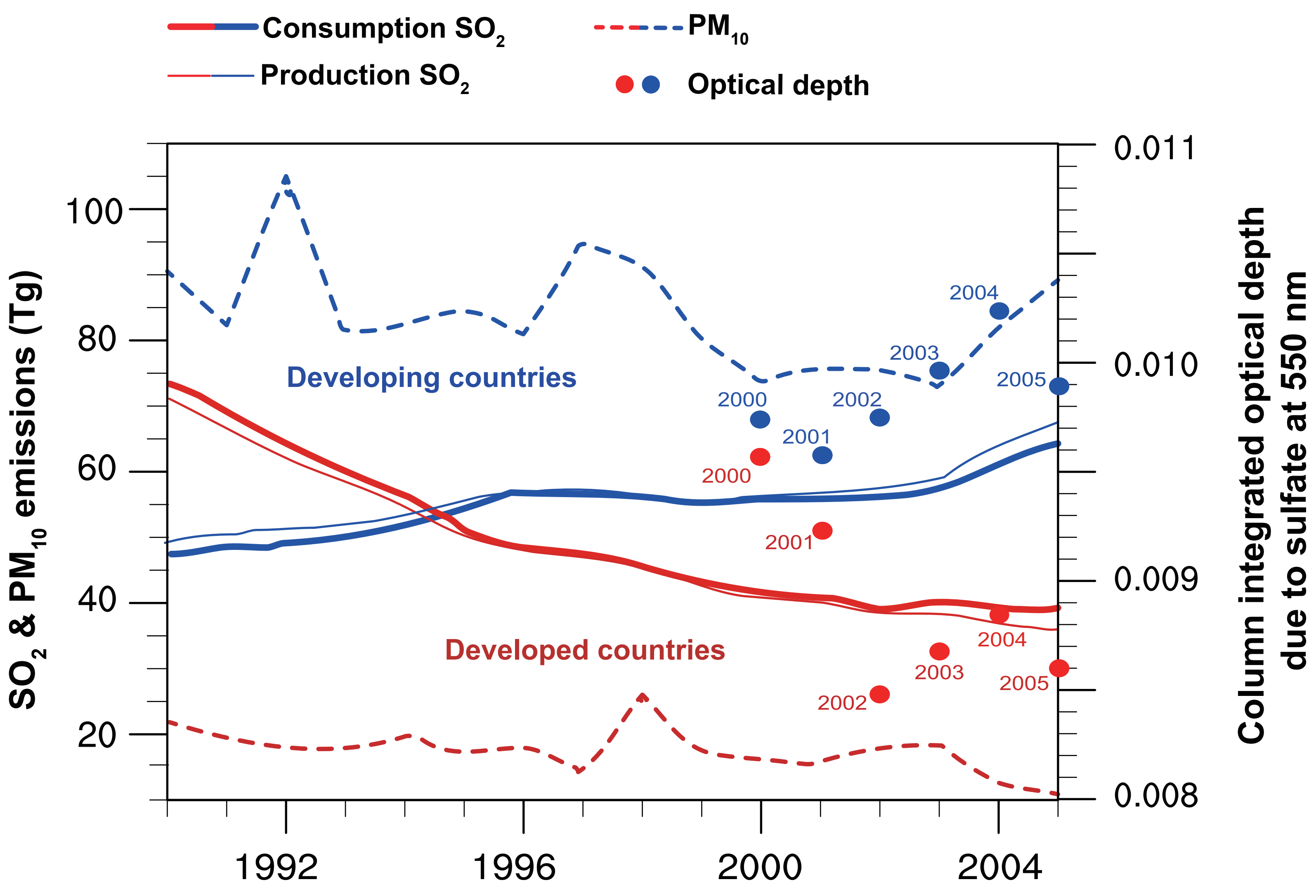

Figure S2. Time series of production-based SO_2 emissions (thin solid lines), consumption-based SO_2 emissions (thick solid lines), column integrated optical depth due to sulfate at 550 nm (dots), and content of PM10 (dash lines) in the developed (red) and the developing (blue) countries.

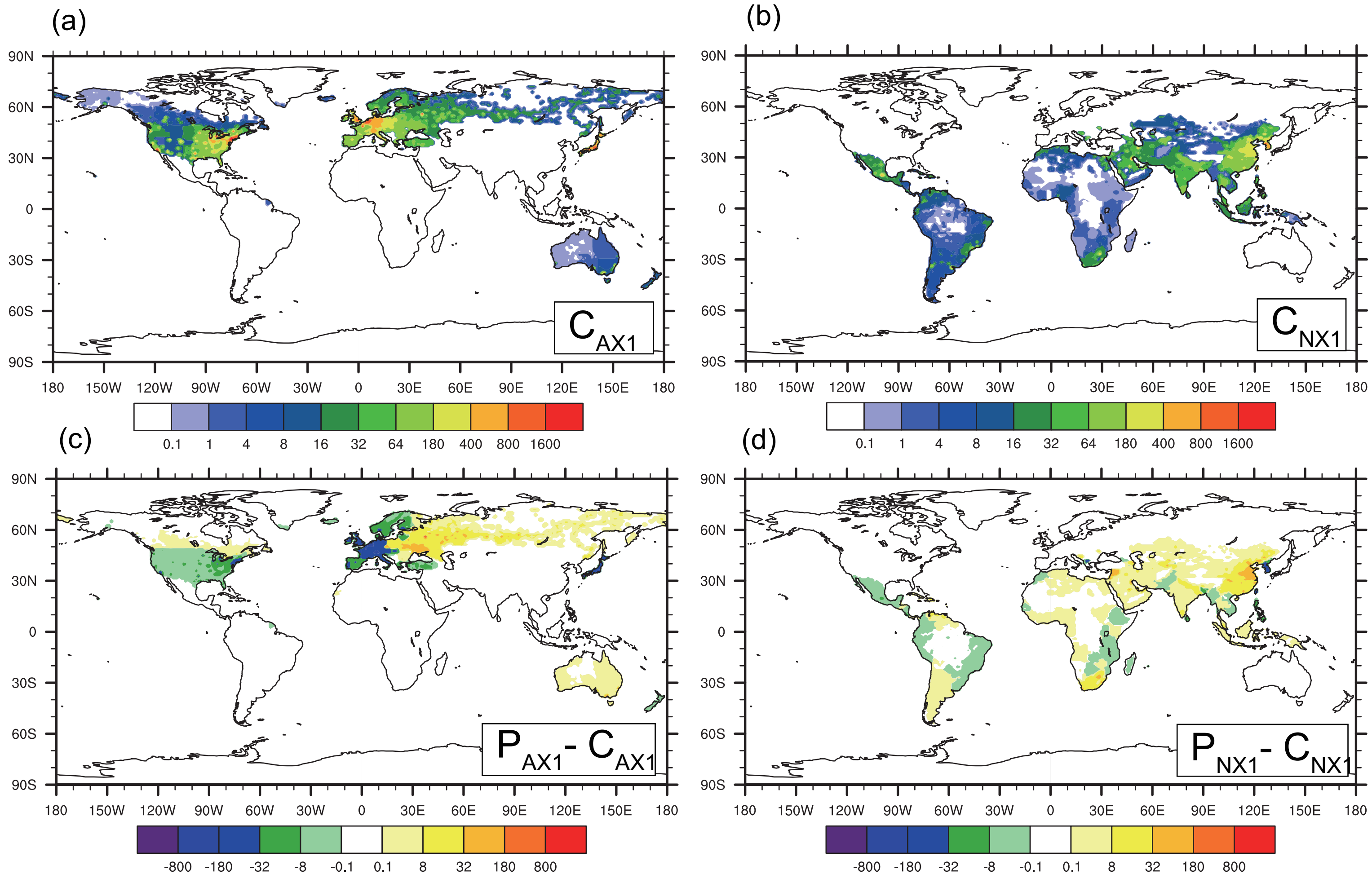

Figure S3. Cumulative CO_2 emission flux (Kg/m^2/year) from 1990 to 2005 for (a) developed world consumption-based emission and (c) its anomalies relative to the production-based emission, and (b) developing world consumption-based emission and (d) its anomalies relative to the production-based emission.The maps were created using NCAR Command Language Version 6.3.0 (http://dx.doi.org/10.5065/D6WD3XH5)[11].

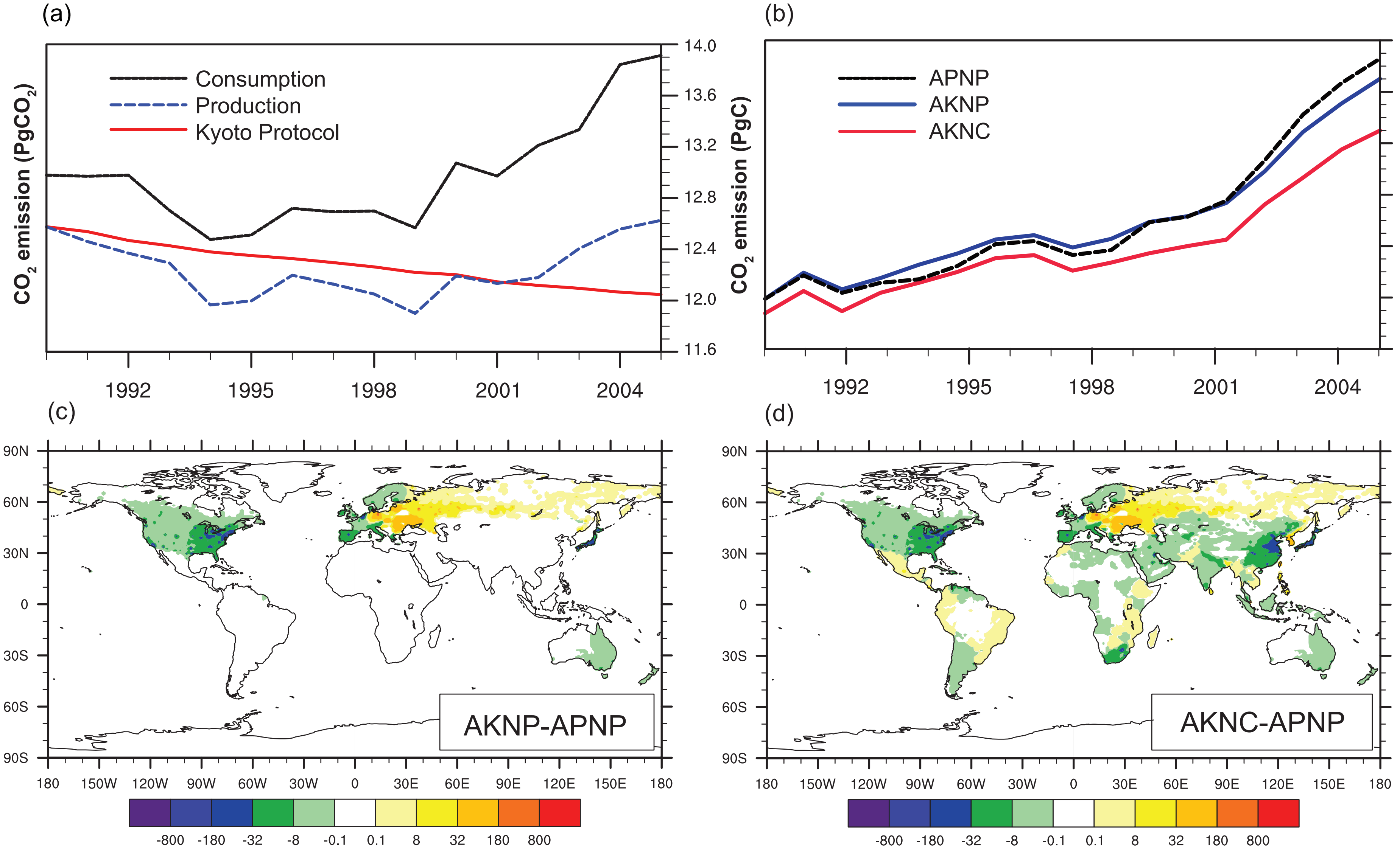

Figure S4. (a) Time series of CO_2 emissions from the developed countries based on production, consumption and KP. (b) Time series of CO_2 emissions under the APNP, AKNP, and AKNC scenarios. (c) Cumulative CO_2 emission flux (Kg/m^2/year) difference between the AKNP and APNP scenarios from 1990 to 2005. (d) Cumulative CO_2 emission flux (Kg/m^2/year) difference between the AKNC and APNP scenarios from 1990 to 2005.The maps were created using NCAR Command Language Version 6.3.0 (http://dx.doi.org/10.5065/D6WD3XH5)[11].
